# Integrating visual assessments and quantification methods for tau PET staging

**DOI:** 10.1002/alz.70352

**Published:** 2025-06-22

**Authors:** Yuna Gu, Jihwan Yun, Alexis Moscoso, Michael Schöll, Daeun Shin, Eun Hye Lee, Heekyong Kang, Sohyun Yim, Hyunjin Jo, Jun Pyo Kim, Sung Hoon Kang, Hee Jin Kim, Duk L. Na, Henrik Zetterberg, Kaj Blennow, Fernando Gonzalez‐Ortiz, Nicholas J Ashton, Hyemin Jang, Michael W. Weiner, Seung Hwan Moon, Hanna Cho, Jae Yong Choi, Kyung Rok Nam, Byung Hyun Byun, Su Yeon Park, Jeong Ho Ha, Soo Hyun Cho, Sang Won Seo, ADNI and K‐ROAD study groups

**Affiliations:** ^1^ Department of Neurology, Samsung Medical Center Sungkyunkwan University School of Medicine, Gangnam‐gu Seoul South Korea; ^2^ Department of Health Sciences and Technology, SAIHST Sungkyunkwan University Seoul South Korea; ^3^ Department of Neurology Kyung Hee University Hospital, Kyung Hee University College of Medicine Seoul South Korea; ^4^ Department of Psychiatry and Neurochemistry, Institute of Neuroscience and Physiology The Sahlgrenska Academy, University of Gothenburg Gothenburg Sweden; ^5^ Wallenberg Centre for Molecular and Translational Medicine University of Gothenburg Gothenburg Sweden; ^6^ Nuclear Medicine Department and Molecular Imaging Group Instituto de Investigación Sanitaria de Santiago de Compostela Santiago de Compostela Spain; ^7^ Dementia Research Centre, Institute of Neurology University College London London UK; ^8^ Department of Neuropsychiatry Sahlgrenska University Hospital Mölndal Sweden; ^9^ Department of Neurology Korea University Guro Hospital, Korea University College of Medicine Seoul South Korea; ^10^ Alzheimer's Disease Convergence Research Center Samsung Medical Center Seoul South Korea; ^11^ Department of Digital Health, SAIHST Sungkyunkwan University Seoul South Korea; ^12^ Department of Intelligent Precision Healthcare Convergence Sungkyunkwan University Suwon South Korea; ^13^ Happymid Clinic Seoul South Korea; ^14^ Department of Psychiatry and Neurochemistry, Institute of Neuroscience and Physiology the Sahlgrenska Academy at the University of Gothenburg Gothenburg Sweden; ^15^ Clinical Neurochemistry Laboratory Sahlgrenska University Hospital Gothenburg Sweden; ^16^ Department of Neurodegenerative Disease UCL Institute of Neurology, Queen Square London UK; ^17^ UK Dementia Research Institute at UCL London UK; ^18^ Hong Kong Center for Neurodegenerative Diseases Clear Water Bay Hong Kong China; ^19^ Wisconsin Alzheimer's Disease Research Center University of Wisconsin School of Medicine and Public Health University of Wisconsin–Madison Madison Wisconsin USA; ^20^ Paris Brain Institute, ICM Pitié‐Salpêtrière Hospital Sorbonne University Paris France; ^21^ Neurodegenerative Disorder Research Center, Division of Life Sciences and Medicine, and Department of Neurology, Institute on Aging and Brain Disorders University of Science and Technology of China and First Affiliated Hospital of USTC Hefei P.R. China; ^22^ Department of Clinical Neuroscience King's College London, Institute of Psychiatry, Psychology and Neuroscience, Maurice Wohl Clinical Neuroscience Institute London UK; ^23^ Department of Clinical Neuroscience, NIHR Biomedical Research Centre for Mental Health and Biomedical Research Unit for Dementia at South London and Maudsley NHS Foundation London UK; ^24^ Centre for Age‐Related Medicine Stavanger University Hospital Stavanger Norway; ^25^ Department of Neurology Seoul National University Hospital, Seoul National University College of Medicine Seoul South Korea; ^26^ Department of Radiology and Biomedical Imaging University of California San Francisco, San Francisco California USA; ^27^ Department of Nuclear Medicine, Samsung Medical Center Sungkyunkwan University School of Medicine Seoul South Korea; ^28^ Department of Neurology Gangnam Severance Hospital Yonsei University College of Medicine Seoul South Korea; ^29^ Division of Applied RI Korea Institute of Radiological & Medical Sciences Seoul South Korea; ^30^ Department of Nuclear Medicine, Korea Cancer Center Hospital Korea Institute of Radiological & Medical Sciences Seoul South Korea; ^31^ Department of Neurology, Korea Cancer Center Hospital Korea Institute of Radiological & Medical Sciences Seoul South Korea; ^32^ Department of Neurology Chonnam National University Medical School and Chonnam National University Hospital Gwangju South Korea

**Keywords:** Alzheimer's disease, amyloid–tau staging, cognitive trajectories, diagnostic precision, plasma biomarkers, phosphorylated tau217, quantitative methods, tau positron emission tomography staging, temporal meta‐region, temporoparietal region, visual assessment

## Abstract

**INTRODUCTION:**

We compared visual assessments and quantification methods for tau positron emission tomography (PET) staging and evaluated plasma biomarkers and cognitive trajectories across amyloid and tau (AT) staging.

**METHODS:**

Tau PET scans from 289 Korea‐Registries to Overcome Dementia and Accelerate Dementia Research (K‐ROAD) participants were analyzed visually and quantitatively, with validation in the Alzheimer's Disease Neuroimaging Initiative (ADNI) cohort (*n* = 870). Plasma biomarkers and cognitive measures were evaluated across AT stages.

**RESULTS:**

FreeSurfer without partial volume correction (PVC) achieved the highest area under the curve (AUC) for tau positivity (0.918), based on visual interpretation. The temporal meta‐region excelled in moderate tau staging (AUC = 0.856), while the temporoparietal region performed best for advanced staging (AUC = 0.828). Quantification methods detected phosphorylated tau217 changes during intermediate transitions (e.g., A+/T− to A+/Tmod+) more effectively. Plasma biomarkers and cognitive measures progressively changed across AT stages, with consistent results in K‐ROAD and ADNI cohorts.

**DISCUSSION:**

The complementary strengths of visual and quantification methods enhance tau PET staging by effectively capturing biomarker changes and cognitive trajectories.

**Highlights:**

- Quantification partially outperformed visual assessments in detecting early tau burdens.
- Meta‐temporal and temporoparietal regions of interest excelled in early and late tau staging.
- Quantification methods correlated with plasma phosphorylated tau217 and cognitive decline.
- Findings were validated across two independent cohorts (Korea‐Registries to Overcome Dementia and Accelerate Dementia Research and Alzheimer's Disease Neuroimaging Initiative).
- The study highlights the complementary roles of visual and quantification methods.

## 1 BACKGROUND

Neurofibrillary tangles, a hallmark of Alzheimer's disease (AD), are composed of hyperphosphorylated tau protein aggregates.^1^ Tau pathology initially emerges in the medial temporal lobe (MTL) and progressively spreads to the inferior and lateral temporal cortices (Braak stage III–IV), eventually extending into the parietotemporal and frontal neocortex (Braak stage V–VI).^2^, ^3^ The advent of tau positron emission tomography (PET) has enabled in vivo visualization of tau deposition, providing a powerful tool for tracking disease progression.^4^, ^5^, ^6^ By capturing the spatial distribution and burden of tau pathology, tau PET aids in distinguishing AD from other neurodegenerative disorders and serves as a critical biomarker for clinical trials targeting disease‐modifying therapies.

Recently, tau PET with ^18^[F]flortaucipir received the US Food and Drug Administration (FDA) and the European Medicines Agency approval for clinical use, providing a standardized approach for staging tau pathology.^7^, ^8^ Validated against neuropathological findings, this visual method classifies tau deposition into negative, moderate, or advanced stages based on tracer uptake in key regions, including the posterolateral temporal, precuneus, occipital, and frontal areas.^5^ While this method effectively identifies cortical tau pathology, subsequent studies have introduced visual assessment approaches that incorporate the MTL, an early site of tau accumulation, to enhance sensitivity for detecting early tau pathology.^9^, ^10^, ^11^

To enhance tau burden measurement in research settings, diverse quantification methods have been developed, including the Mayo Clinic Adult Lifespan Template (MCALT)^12^ based on Statistical Parametric Mapping (SPM)^13^ and FreeSurfer with^14^, ^15^ or without partial volume correction (PVC).^16^, ^17^ Additionally, various regions of interest (ROIs), such as the MTL, temporal meta‐region (meta‐temporal), neocortical temporal (neo‐temporal), and temporoparietal regions, have been explored to better capture the progression of tau pathology. However, further investigation is needed to determine which quantification method and ROI selection most closely align with the FDA‐approved visual assessment approach commonly used in clinical settings.^7^, ^9^, ^11^

Tau PET is widely recognized as a key T2 biomarker for staging and prognosis in AD. Given the established staging framework, AD biomarker levels, including plasma phosphorylated‐tau 217 (p‐tau217), which reflects amyloid and tau (AT) pathology, as well as non‐specific AD‐related biomarkers such as glial fibrillary acidic protein (GFAP) and neurofilament light chain (NfL), are expected to vary across different tau stages.^18^, ^19^, ^20^ Additionally, prognosis is likely to differ according to tau staging. However, whether tau staging based on visual assessments or quantification methods across different ROIs more accurately reflects AD biomarker levels and prognosis remains unclear. Additionally, investigating the potential complementarity of these approaches may offer further insights into their respective roles in disease characterization.

In this study, we compared the FDA‐approved visual approach^8^ and several quantification methods for tau PET staging in two independent cohorts to assess their clinical utility. Specifically, we aimed to: (1) identify which of three quantitative methods (MCALT, and FreeSurfer with/without PVC) best align with visual assessments, (2) determine the most consistent ROIs (MTL, meta‐temporal, neo‐temporal, and temporoparietal regions) for visual tau staging, and (3) evaluate plasma biomarker levels and cognitive trajectories across tau staging defined by visual and quantitative assessments.

## 2 METHODS

### 2.1 Participants

A total of 289 individuals were prospectively recruited from the Korea‐Registries to Overcome Dementia and Accelerate Dementia Research (K‐ROAD) cohort,^21^ which is a genotype–phenotype cohort to accelerate the development of innovative diagnostic and therapeutic techniques for neurodegenerative diseases, mainly AD and related dementia syndromes, in collaboration with 25 nationwide university‐affiliated hospitals in South Korea. These participants underwent [^18^F]flortaucipir PET scans between May 2015 and December 2023. Each participant also underwent neuropsychological assessments, brain magnetic resonance imaging (MRI), and amyloid beta (Aβ) [^18^F]florbetaben or [^18^F]flutemetamol PET scans. Additionally, external validation was conducted using data from the Alzheimer's Disease Neuroimaging Initiative (ADNI), comprising 870 participants who underwent similar neuropsychological assessments, tau PET imaging, and MRI scans. When tau PET data alone were analyzed, we included all 870 available ADNI cases. However, when amyloid PET status needed to be considered alongside tau PET, we included only those cases for which amyloid PET status at the time of tau PET could be determined. Specifically, we determined Aβ status for 518 individuals who (1) underwent Aβ PET within 1 year of the tau PET scan, (2) were Aβ positive (≥ 1.11 standardized uptake value ratio [SUVR]) prior to the tau PET scan, or (3) were Aβ negative after the tau PET scan. The remaining 352 participants did not meet these time or data availability criteria, and thus their Aβ status could not be definitively established for this analysis.

**RESEARCH IN CONTEXT**

**Systematic Review**: The authors performed a systematic review of the literature using PubMed, focusing on studies that examined visual assessments and quantification methods for tau positron emission tomography (PET) staging. Previous research predominantly used quantification methods for tau burden measurement and staging. However, direct comparisons between visual assessments and quantification methods remain limited, and few studies have explored their combined utility in evaluating plasma biomarkers and cognitive trajectories across amyloid and tau (AT) stages.

**Interpretation**: This study highlights the superior sensitivity of quantification methods, particularly FreeSurfer without partial volume correction (PVC), in detecting early and intermediate tau pathology, capturing nuanced differences in phosphorylated tau217 levels and cognitive trajectories. While visual assessments excel in diagnosing advanced stages, consistent findings across Korea‐Registries to Overcome Dementia and Accelerate Dementia Research and Alzheimer's Disease Neuroimaging Initiative cohorts emphasize the complementary roles of visual assessments for simplicity and quantification methods for precision, advancing tau PET staging.

**Future Directions**: Future research should refine quantification pipelines, focusing on PVC techniques and region of interest selection, and apply combined visual and quantification methods to diverse cohorts. Integrating plasma biomarkers with tau PET in longitudinal studies and investigating visual‐quantification discordances could enhance diagnostics and therapy monitoring.

According to the National Institute on Aging–Alzheimer's Association (NIA‐AA) research framework's syndrome cognitive staging, participants were categorized into three groups: cognitively unimpaired (CU), mild cognitive impairment (MCI), and dementia.^22^ The CU group was defined by the following criteria: (1) absence of any medical history likely to impact cognitive function based on Christensen's health screening criteria,^23^, ^24^ and (2) no objective cognitive impairment as measured by a comprehensive neuropsychological test battery across all cognitive domains (scores above −1.0 standard deviation [SD]) of age‐ and education‐adjusted norms in memory and −1.5 SD in other cognitive domains).^25^ Participants classified as MCI met the following criteria^26^: (1) subjective cognitive complaints from either the participant or their caregiver, (2) objective cognitive impairment in any domain (scores below −1.0 SD of age‐ and education‐adjusted norms in memory and −1.5 SD in other cognitive domains), (3) no significant difficulties in activities of daily living, and (4) absence of dementia. Participants with clinically diagnosed AD dementia (AD‐D) met the NIA‐AA diagnostic criteria.^27^ The criteria for diagnosis with subcortical vascular cognitive impairment (SVCI), which encompasses subcortical vascular MCI and subcortical vascular dementia were as follows: (1) subjective cognitive complaints by the patient or caregiver; (2) objective cognitive impairment below −1.0 SD in any domain (including language, visuospatial, memory, or frontal function) upon neuropsychological testing; and (3) severe ischemia identified on brain MRI, defined as the presence of periventricular white matter hyperintensities (WMHs) > 10 mm and deep WMH > 25 mm, as modified from the Fazekas ischemia criteria.^28^ Moreover, participants with frontotemporal dementia (FTD) syndromes included those with a clinical diagnosis of behavioral variant FTD (bvFTD), semantic variant primary progressive aphasia (svPPA), non‐fluent/agrammatic variant PPA (nfvPPA), corticobasal syndrome (CBS), progressive supranuclear palsy syndrome (PSPS), or FTD–motor neuron disease (MND). Probable bvFTD was clinically defined based on the criteria outlined by Rascovsky et al.,^29^ whereas svPPA and nfvPPA were diagnosed based on the criteria provided by Gorno‐Tempini et al.^30^ Furthermore, CBS,^31^ PSPS,^32^ and FTD‐MND^33^ were diagnosed according to each diagnostic criteria. All FTD syndromes were diagnosed based on the patient's clinical course, neurologic examination, neuropsychological testing, and brain imaging.

We obtained written informed consent for the K‐ROAD study, and the institutional review board of each participating center approved the study protocol. Additionally, the ADNI Data Sharing and Publications Committee approved data use and publication.

### 2.2 MRI data acquisition

All participants underwent 3D T1 turbo field echo imaging with sagittal slice thickness of 1.0 mm with a 50% overlap, no gap, a repetition time (TR) of 9.9 ms, an echo time (TE) of 4.6 ms, a flip angle of 8°, and a matrix size of 240 × 240 pixels, reconstructed to 480 × 480 over a field of view of 240 mm.^34^

### 2.3 Aβ PET acquisition and definition of Aβ positivity

All Korean participants underwent one of the following Aβ PET scans: florbetaben or flutemetamol. For florbetaben and flutemetamol PET, a 20 minute dynamic emission PET scan (divided into four 5 minute segments) was performed 90 minutes after administration of a mean dose of 311.5 MBq of florbetaben or 197.7 MBq of flutemetamol. The resulting three‐dimensional PET images were reconstructed using the ordered‐subset expectation maximization algorithm, resulting in a 128 × 128 × 48 matrix with voxel dimensions of 2 × 2 × 3.27 mm (florbetaben: 4 iterations, 20 subsets; flutemetamol: 4 iterations, 20 subsets). Aβ uptake was measured using a regional direct comparison Centiloid (rdcCL) method developed in our previous study, which harmonizes florbetaben and flutemetamol PET ligands without the need for ^11^C‐labelled Pittsburgh compound B^35^ images. Aβ PET positivity (A+) was defined using a global MRI‐based rdcCL threshold of 25.5, which was obtained using the Gaussian mixture model from 3753 participants aged ≥ 55 years who underwent florbetaben or flutemetamol PET.^36^, ^37^ The florbetaben and flutemetamol global MRI‐based rdcCL scales showed an area under the curve (AUC) > 0.9 for Aβ PET positivity by visual assessment. All imaging analyses for the K‐ROAD study were conducted at the laboratory of Samsung Medical Center (SMC), which served as a core center.

All ADNI participants underwent [^18^F]florbetapir PET scans, and SUVRs were obtained from the ADNI dataset. Previous studies^38^, ^39^, ^40^ have used a cut‐off of 1.11 SUVR for ADNI—calculated using the cerebellar cortex as the reference region—which converts to 19.8 CL using the equation CL = 188.22 × SUVR_florbetapir _− 189.16.^38^, ^39^

### 2.4 Tau PET data acquisition

The flortaucipir PET images were acquired using two different PET/CT (computed tomography) scanners: a Discovery STE PET/CT (GE Healthcare) at SMC and a Biograph mCT PET/CT scanner (Siemens Medical Solutions) at Gangnam Severance Hospital. Each participant received an intravenous bolus injection of ≈ 280 MBq of flortaucipir. PET images were then acquired over a 20 minute period starting 80 minutes post‐injection. To minimize head movement during the scan, a head holder was used, and brain CT images were acquired beforehand for attenuation correction. The 3D PET images were reconstructed using the OSEM algorithm (iterations = 6, subsets = 16) with a matrix size of 128 × 128 × 47 and a voxel size of 2.00 × 2.00 × 3.27 mm at SMC, and a matrix size of 256 × 256 × 223 with a voxel size of 1.591 × 1.591 × 1 mm at Gangnam Severance Hospital. In ADNI, all participants underwent tau PET with flortaucipir. Details of ADNI PET acquisition and processing have been described in previously published studies.^41^, ^42^, ^43^, ^44^

### 2.5 Tau PET visual assessment method

Following FDA‐approved guidelines for flortaucipir PET interpretation, tau PET scans were visually interpreted using a two‐step process comprising image preparation and image interpretation, following the same approach in both the K‐ROAD and ADNI cohorts.^5^, ^8^ First, the reconstructed Tau PET images were co‐registered to T1‐weighted MRIs using SPM12^13^ and reoriented to eliminate head tilt. During image preparation, a color scale was applied based on the cerebellar reference: background activity was defined as up to 1.65 times the cerebellar average, following the standard protocol. Next, according to FDA‐approved guidelines,^8^ a tau PET scan was classified as negative if there was no increased neocortical uptake or if uptake was confined to the mesial temporal, anterolateral temporal, or frontal regions. A moderate AD tau pattern showed increased uptake in the posterolateral temporal or occipital region, while an advanced pattern extended to the parietal/precuneus or frontal region. In K‐ROAD, three trained neurologists (E.H.L., D.S., J.Y.), who were blinded to other clinical information, independently rated the images. Majority reads were assigned when three assessments were available, with the final rating based on the majority decision. Inter‐reader agreement was evaluated using Fleiss κ, yielding a κ of 0.75, consistent with previously reported values (0.74–0.89) for the FDA‐approved flortaucipir PET visual method.^7^, ^8^ For the ADNI dataset, visual assessments of [^18^F]flortaucipir PET images were carried out by an experienced reader (A.M.).

### 2.6 Quantitative analysis methods for flortaucipir PET

To calculate tau PET SUVRs, we used three accepted methods: MCALT, FreeSurfer without PVC, and FreeSurfer with PVC. These methods provide complementary approaches to analyzing tau deposition in the brain. For the first method, SUVRs were calculated using the MCALT, which is specifically designed for aging and AD populations. The tau PET images were co‐registered to structural MRI scans and then non‐linearly registered to MCALT space using Advanced Normalization Tools (https://www.nitrc.org/projects/mcalt/). Using the inferior cerebellar gray matter as the reference region, SUVR values were derived from predefined ROIs registered from the MCALT atlas to native space. For the FreeSurfer method without PVC, FreeSurfer 6.0 (http://surfer.nmr.mgh.harvard.edu/) was used to generate ROI masks in native space, with the inferior cerebellar gray matter as the reference region. The method without PVC was included to provide a baseline for comparison, reflecting the variability commonly observed in PET analyses. In contrast, FreeSurfer with PVC used the region‐based voxel‐wise method through the PET PVC toolbox^45^ to correct for spillover effects from adjacent regions, improving the accuracy of SUVR measurements.

SUVR values were calculated for four key brain regions: the MTL ROI, which included the entorhinal cortex and amygdala; and the meta‐temporal ROI, which exhibited slight variation depending on the method used. In accordance with the MCALT method, the meta‐temporal ROI encompassed the entorhinal cortex, amygdala, fusiform gyrus, parahippocampal gyrus, and inferior and middle temporal cortices.^4^, ^46^, ^47^, ^48^ In the FreeSurfer method, the meta‐temporal ROI encompasses the entorhinal cortex, amygdala, fusiform gyrus, parahippocampal gyrus, inferior temporal cortex, and lingual gyrus. Thus, the meta‐temporal ROI broadly covered early tau regions (e.g., entorhinal cortex and hippocampus, corresponding to Braak stages I–II) as well as adjacent temporal areas involved in subsequent tau propagation. The neo‐temporal ROI was focused on the inferior and middle temporal cortices, while the temporoparietal ROI included the inferior, middle, and superior temporal cortices, as well as the superior parietal gyrus, inferolateral parietal lobe, and posterior cingulate gyrus.^4^, ^48^, ^49^

Tau positivity in each ROI was defined using cutoffs calculated as the SUVR mean + 2 SD derived from an Aβ‐negative CU reference group, a widely validated approach providing high specificity.^24^, ^47^, ^50^ Quantitative tau stages (“Mod [Tmod+]” and “Adv [Tadv+]”) were subsequently defined in each ROI using receiver operating characteristic (ROC)‐derived cut‐points, with visual assessments serving as the reference standard to empirically optimize the separation between moderate and advanced stages based on visual staging criteria.

### 2.7 Plasma collection and processing

The detailed plasma collection and processing methods are described in Supplementary Material 1 in supporting information. Plasma concentrations of p‐tau217, GFAP, and NfL were measured using single molecule array assays at the University of Gothenburg and DNALINK. GFAP and NfL concentrations were quantified using the commercial Neurology 4‐Plex E kit (Quanterix). The concentration of p‐tau217 was measured using the commercial ALZpath p‐tau217 assay kit. The measurements were performed by analysts blinded to the clinical data from one round of experiments using one batch of reagents. The intra‐assay coefficients of variation of the biomarker assays were < 10%. We used the publicly accessible ADNI cohort. From the ADNI database, we analyzed plasma samples from 378 participants (CU, *n* = 209; MCI, *n* = 148; AD‐D, *n* = 21). Plasma biomarkers were measured using two different assay platforms: p‐tau217 was quantified using Alzpath assays, while GFAP and NfL were measured using the commercial Neurology 4‐Plex Ekit (Quanterix). All plasma biomarker data were obtained from the ADNI database (filename: All_Subjects_FNIHBC_Blood_BIOMARKER_TRAJECTORIES_10Oct2024). The intra‐assay coefficients of variation of the biomarker assay were < 10%.

### 2.8 Longitudinal follow‐up

Participants with non‐AD diagnoses (SVCI or FTD) were excluded from longitudinal analyses to ensure a homogeneous AD‐focused sample. For the longitudinal analyses, only participants diagnosed as CU, MCI, or AD‐D were included in both cohorts. Specifically, in the K‐ROAD cohort, 164 participants with two or more Mini‐Mental State Examination (MMSE) assessments were included in the longitudinal MMSE analysis, and 140 participants with repeated Clinical Dementia Rating‐Sum of Boxes (CDR‐SB) assessments were included in the CDR‐SB analysis.^52^ Retrospective and prospective MMSE and CDR‐SB scores were obtained relative to the time of tau PET scans, with a mean follow‐up of 3.20 years (range 0.5–9.3) for MMSE and 2.73 years (range 0.6–9.3) for CDR‐SB analyses. From ADNI, 374 participants were included in the MMSE analysis and 386 in the CDR‐SB analysis, with a mean follow‐up of 2.73 years (range 0.7–7.7) for MMSE and 2.82 years (range 0.7–7.7) for CDR‐SB. A detailed flowchart clearly illustrates the participant inclusion/exclusion process (Figure S1 in supporting information).

### 2.9 Statistical analyses

A comparison of SUVRs between groups was conducted using linear regression models, with adjustments made for age and apolipoprotein E (*APOE*) genotype. ROC analyses were conducted to evaluate the efficacy of tau quantitative assessment in predicting tau positivity and tau PET stages defined by visual assessment. The AUC of each method for predicting tau positivity was compared using the DeLong test. The agreement between the visual assessment and quantitative assessment was studied using the Cohen kappa (κ) statistic. Due to the non‐normality of the data, plasma biomarker analyses were performed after log transformation. The differences in plasma biomarker levels between the groups were assessed using analysis of covariance (ANCOVA), with age and *APOE* genotypes as covariates, followed by Tukey‐corrected post hoc pairwise comparisons. To investigate the effects of AT staging on cognitive trajectories, a linear mixed‐effects model with random intercepts and random slopes of time was conducted in each AT stage, after adjusting for age and *APOE* genotype as covariates (fixed effects: group, time, group x time, covariates). To correct for multiple comparisons, a false discovery rate (FDR) of 0.05 was used. Specifically, FDR correction was applied to the multiple group comparisons following the linear mixed model (LMM) analyses presented in cognitive trajectories by visual and ROI‐based quantitative tau staging in K‐ROAD and ADNI cohorts. All statistical analyses were conducted using R version 4.3.1, with a significance threshold of *P* < 0.05.

## 3 RESULTS

### 3.1 Participant demographics

Table 1 presents participant demographics. The K‐ROAD cohort (*n* = 289, mean age 72.4 ± 9.7 years, 60.6% female, 42.9% *APOE* ε4 carriers) consisted of CU (12.8%), MCI (18.0%), AD‐D (29.4%), SVCI (27.7%), and FTD (12.1%) participants. Mean global Aβ uptake was 64.2 ± 56.1 CL, with 64.0% Aβ‐positive cases. The ADNI cohort (*n* = 870, mean age 71.0 ± 7.1 years, 52.0% female, 34.0% *APOE* ε4 carriers) included CU (55.3%), MCI (33.2%), and AD‐D (11.5%) participants. Mean global Aβ uptake was 33.6 ± 43.7 CL, with 46.4% Aβ‐positive cases.

**TABLE 1:** Demographics of study participants.

	K‐ROAD	ADNI
	(*N* = 289)	(*N* = 870)
Age, years	72.4 ± 9.7	71.0 ± 7.1
Sex, female, *n* (%)	175 (60.6%)	452 (52.0%)
Education, years	10.6 ± 5.1	16.4 ± 2.4
*APOE* ε4 carriers, *n (%)*	123 (42.9%)	295 (34.0%)
Diagnosis, CU/MCI/AD‐D/SVCI/FTD, *n* (%)	37/52/85/80/35 (12.8/18.0/29.4/27.7/12.1)	481/289/100/0/0 (55.3/33.2/11.5/0/0)
Aβ uptake (Centiloid)	64.2 ± 56.1^a^	33.6 ± 43.7
Amyloid positivity, *n* (%)	185 (64.0%)^b^	240 (46.4%)^c^
MMSE	20.1 ± 7.6	27.8 ± 3.2
CDR‐SB	4.6 ± 4.2	1.1 ± 2.0

Abbreviations: AD‐D, Alzheimer's disease dementia; ADNI, Alzheimer's Disease Neuroimaging Initiative; *APOE*, apolipoprotein E; Aβ, amyloid beta; CDR, Clinical Dementia Rating; CDR‐SB, Clinical Dementia Rating Sum of Boxes; CL, Centiloid; CU, cognitively unimpaired; FTD, frontotemporal dementia; K‐ROAD, Korea‐Registries to Overcome Dementia and Accelerate Dementia; MCI, mild cognitive impairment; MMSE, Mini‐Mental State Examination; PET, positron emission tomography; SUVR, standardized uptake value ratio; SVCI, subcortical vascular cognitive impairment.

^a^ Aβ uptake was measured using a regional direct comparison Centiloid (rdcCL) method developed in our previous study, which harmonizes ^18^F‐florbetaben and ^18^F‐flutemetamo PET ligands without the need for ^11^C‐labelled Pittsburgh compound B images.

^b^ Aβ PET positivity threshold was set at a global regional direct comparison Centiloid value of 25.5 using ^18^F‐florbetaben and ^18^F‐flutemetamol tracers.

^c^ Amyloid PET positivity threshold was set at a Klunk Centiloid value of 19.8 (equivalent to SUVR 1.11) using the ^18F^‐florbetapir tracer.

### 3.2 Comparison of several quantitative assessments against visual assessment for tau positivity in K‐ROAD

**FIGURE 1:**
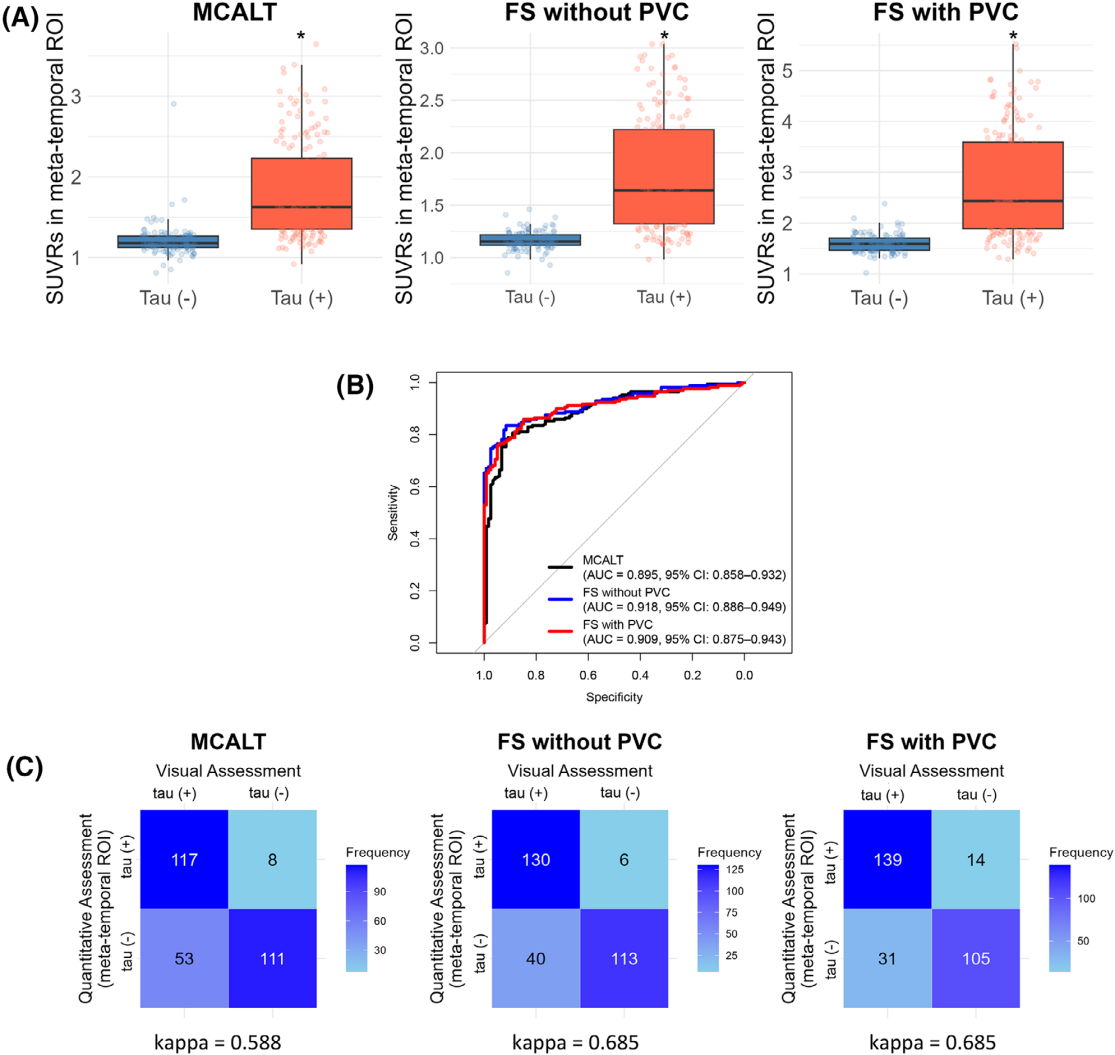
Quantification method comparison for tau positivity. A, SUVR distributions for tau PET‐positive (+) and tau PET‐negative (−) groups across MCALT, FS without PVC, and FS with PVC for K‐ROAD. B, ROC curves comparing diagnostic performance of each method for K‐ROAD. C, Confusion matrices showing agreement between methods and visual assessment for tau positivity for K‐ROAD. FS, FreeSurfer; K‐ROAD, Korea‐Registries to Overcome Dementia and Accelerate Dementia Research; MCALT, Mayo Clinic Alzheimer's Template; PET, positron emission tomography; PVC, partial volume correction; ROC, receiver operating characteristic; ROI, region of interest; SUVR, standardized uptake value ratio.

The SUVR values in the meta‐temporal region were significantly higher in the visually tau‐positive group compared to the tau‐negative group across all three quantitative assessments (*P* < 0.001) (Figure 1A). ROC curves for all quantitative assessments, based on tau positivity defined by visual assessment, were calculated (Figure 1B). Among the quantitative assessments, the FreeSurfer without PVC showed the highest performance, achieving an AUC of 0.918 (95% confidence interval [CI]: 0.886–0.949), although the performance was not significantly higher compared to the other methods (FreeSurfer without PVC vs. MCALT, *P* value = 0.090; FreeSurfer without PVC vs. FreeSurfer with PVC, *P* value = 0.334). To assess agreement between visual and quantitative assessments for tau positivity, Cohen Kappa statistics were calculated (Figure 1C). The quantitative assessments derived from the FreeSurfer method exhibited the highest agreement with visual assessment, with a Kappa value of 0.685. Notably, applying PVC to the FreeSurfer method did not alter the level of agreement, as the Kappa value remained at 0.685.

### 3.3 Quantitative tau uptake, diagnostic performance, and agreement with visual tau staging across ROIs

**FIGURE 2:**
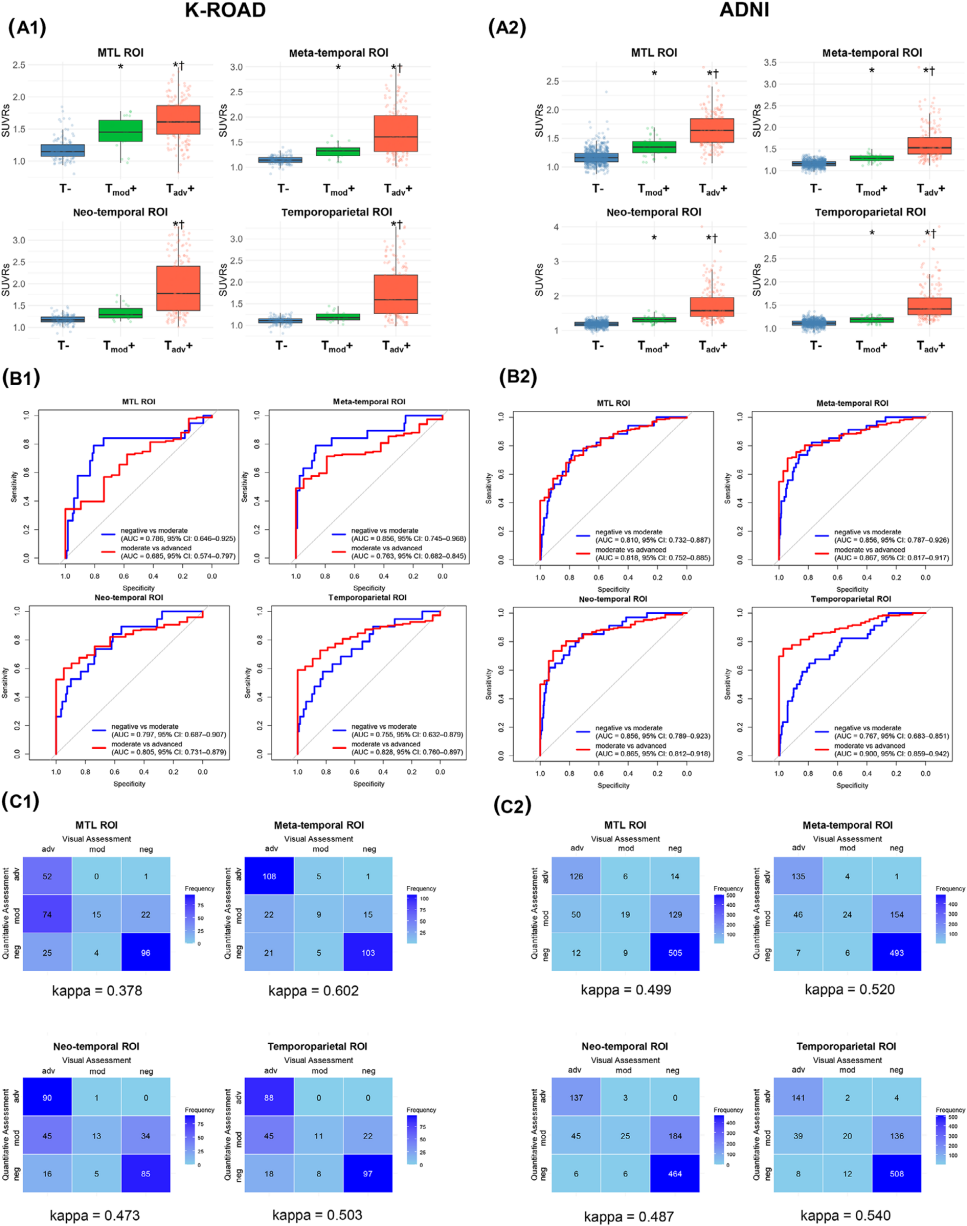
Quantitative tau uptake (A), diagnostic performance (B), and agreement (C) with visual tau staging across ROIs in K‐ROAD (A1,B1,C1) and ADNI cohorts (A2,B2,C2). In visual assessments, Neg (T−) indicates a negative tau pattern, Mod (Tmod+) a moderate tau pattern (posterolateral temporal or occipital uptake), and Adv (Tadv+) an advanced tau pattern (parietal/precuneus or frontal uptake). Quantitative positivity in each ROI was defined as SUVR mean + 2 SD from an amyloid‐negative CU reference group, with staging determined using ROI‐specific ROC‐derived cutoffs based on visual assessments. Inter‐group comparison: ^*^ *P* < 0.05 compared to negative group, **^†^** *P* < 0.05 compared to moderate group. ADNI, Alzheimer's Disease Neuroimaging Initiative; AUC, area under the curve; CU, cognitively unimpaired; K‐ROAD, Korea‐Registries to Overcome Dementia and Accelerate Dementia Research; MTL, medial temporal lobe; ROC, receiver operating characteristic; ROI, region of interest; SD, standard deviation; SUVR, standardized uptake value ratio.

Given its highest agreement with visual tau positivity, subsequent analyses used the FreeSurfer without PVC method. In both K‐ROAD and ADNI cohorts, SUVRs consistently increased with advancing visual tau stages across all ROIs, although statistical significance was not observed in the neo‐temporal ROI and temporoparietal ROIs in K‐ROAD (Figure 2A1 and 2A2). SUVR in the meta‐temporal ROI best differentiated negative from moderate visual tau stages in K‐ROAD (AUC: 0.856, 95% CI: 0.745–0.968) and ADNI (AUC: 0.856, 95% CI: 0.787–0.926; Figure 2B1 and 2B2). SUVR in the temporoparietal ROI best distinguished moderate from advanced stages in both cohorts, with AUCs of 0.828 (95% CI: 0.760–0.897) in K‐ROAD and 0.900 (95% CI: 0.859–0.942) in ADNI. Cohen Kappa statistics were calculated to evaluate agreement between visual assessments and semi‐quantitative tau staging (“Mod [Tmod+]” and “Adv [Tadv+]”), which were defined in each ROI using ROC‐derived cutoffs based on visual assessment as the reference standard. The meta‐temporal and temporoparietal ROIs demonstrated the strongest concordance between quantitative and visual tau staging, with slight variations in concordance strength between two cohorts (Figure 2C1 and 2C2).

### 3.4 AD plasma biomarker levels by visual and ROI‐based quantitative tau staging

**FIGURE 3:**
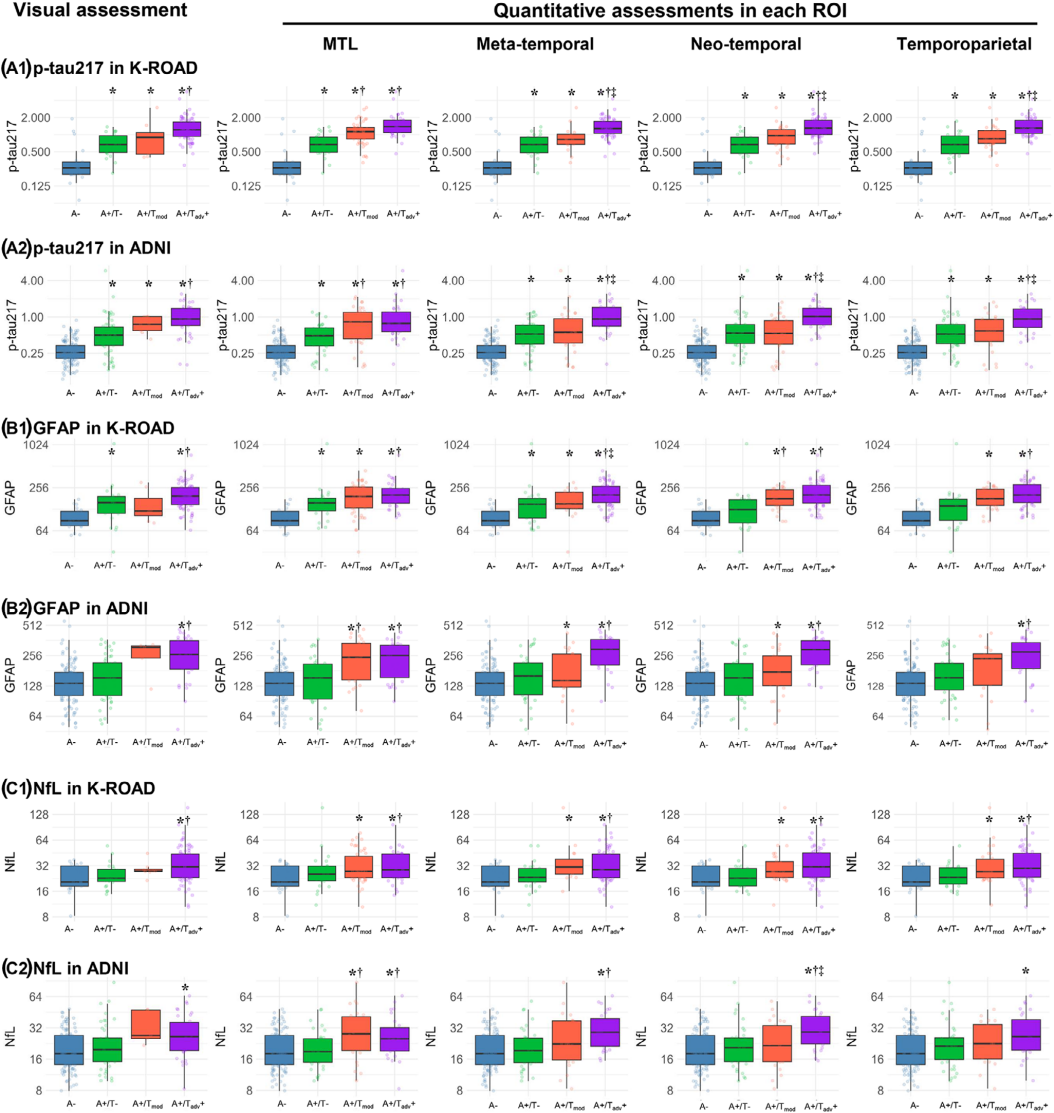
AD plasma biomarker levels including p‐tau217 (A), GFAP (B), and NfL (C) by visual and ROI‐based quantitative tau staging in K‐ROAD (A1,B1,C1) and ADNI cohorts (A2,B2,C2). In visual assessments, Neg (T−) indicates a negative tau pattern, Mod (Tmod+) a moderate tau pattern (posterolateral temporal or occipital uptake), and Adv (Tadv+) an advanced tau pattern (parietal/precuneus or frontal uptake). Quantitative positivity in each ROI was defined as SUVR mean + 2 SD from an amyloid‐negative CU reference group, with staging determined using ROI‐specific ROC‐derived cutoffs based on visual assessments. Inter‐group comparison: ^*^ *P* < 0.05 compared to A– group, **^†^** *P* < 0.05 compared to A+/T– group, **^‡^** *P* < 0.05 compared to A+/T_mod_– group. AD, Alzheimer's disease; ADNI, Alzheimer's Disease Neuroimaging Initiative; CU, cognitively unimpaired; GFAP, glial fibrillary acidic protein; K‐ROAD, Korea‐Registries to Overcome Dementia and Accelerate Dementia Research; NfL, neurofilament light chain; p‐tau, phosphorylated tau; ROC, receiver operating characteristic; ROI, region of interest; SD, standard deviation; SUVR, standardized uptake value ratio.

As shown in Figure 3, levels of plasma biomarkers (p‐tau217, GFAP, and NfL) increased progressively with advancing visual and ROI‐based quantitative tau staging for both K‐ROAD and ADNI cohorts. Notably, for p‐tau217, visual tau staging differentiated only A+/T− from A+/Tadv+, whereas ROI‐based quantitative tau staging distinguished not only A+/T− from A+/Tadv+, but also A+/T− from A+/Tmod+ (MTL ROI) and A+/Tmod+ from A+/Tadv+ (meta‐temporal, neo‐temporal, and temporoparietal ROIs; Figure 3A). For GFAP (Figure 3B) and NfL (Figure 3C), both cohorts demonstrated consistent patterns, with significant differences primarily between A+/T− and A+/Tadv+ in both visual assessment and quantification methods (Table S1 in supporting information).

### 3.5 Cognitive trajectories by visual and ROI‐based quantitative tau staging

**FIGURE 4:**
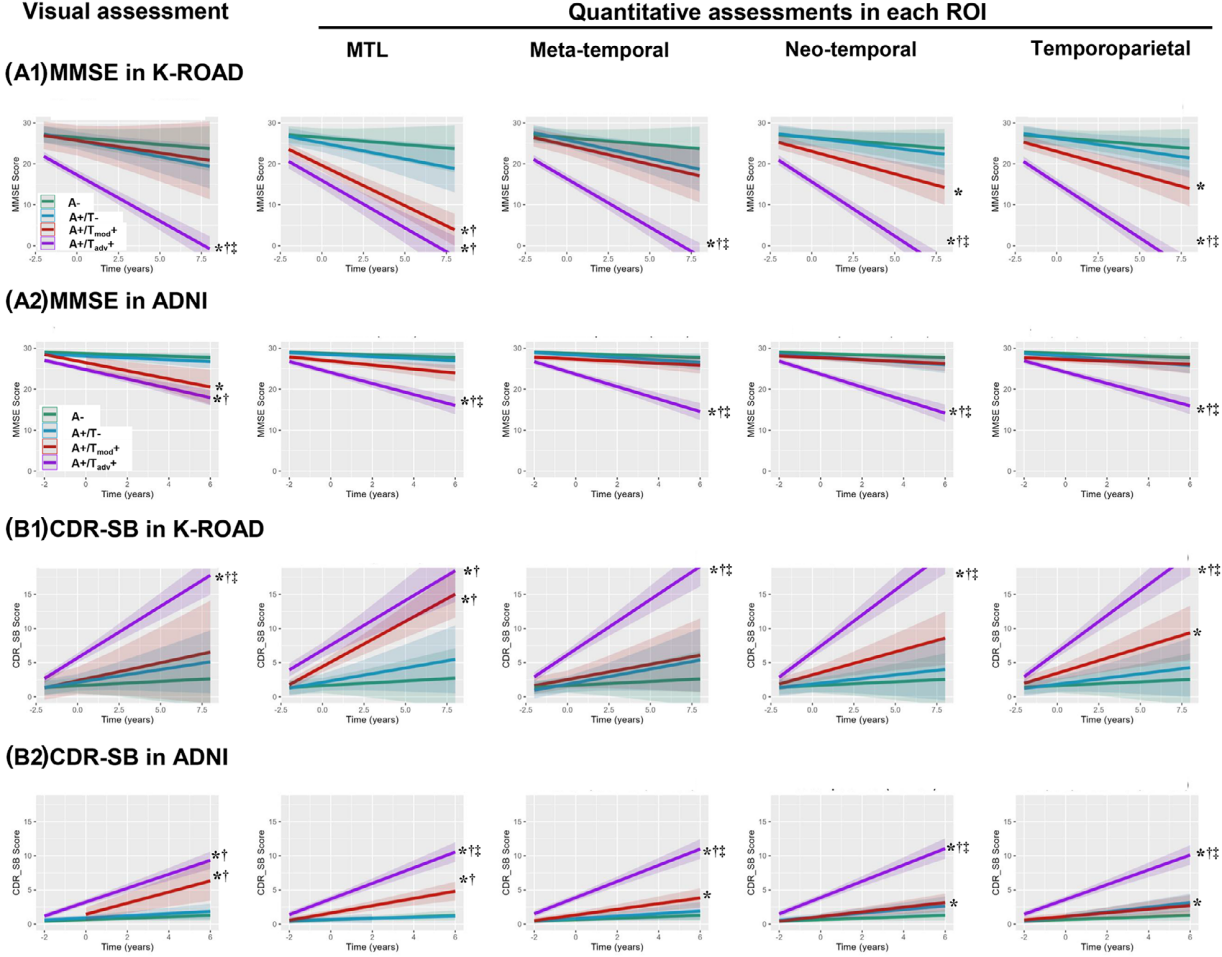
Cognitive trajectories including MMSE (A) and CDR‐SB (B) by visual and ROI‐based quantitative tau staging in K‐ROAD (A1, B1) and ADNI (A2, B2) cohorts. In visual assessments, Neg (T−) indicates a negative tau pattern, Mod (Tmod+) a moderate tau pattern (posterolateral temporal or occipital uptake), and Adv (Tadv+) an advanced tau pattern (parietal/precuneus or frontal uptake). Quantitative positivity in each ROI was defined as SUVR mean + 2SD from an amyloid‐negative CU reference group, with staging determined using ROI‐specific ROC‐derived cutoffs based on visual assessments. Inter‐group comparison: ^*^ *p* < 0.05 compared to A‐ group, **^†^** *p* < 0.05 compared to A+/T‐ group, **^‡^** *p* < 0.05 compared to A+/T_mod_‐ group. A, amyloid; ADNI, Alzheimer's Disease Neuroimaging Initiative; AUC, area under the curve; CDR‐SB, Clinical Dementia Rating Scale‐Sum of Boxes; K‐ROAD, Korea‐Registries to Overcome Dementia and Accelerate Dementia Research; MMSE, Mini‐Mental State Examination; MTL, medial temporal lobe; ROC, receiver operating characteristic; T, tau.

In both K‐ROAD and ADNI cohorts, cognitive declines, as measured by MMSE and CDR‐SB, accelerated progressively with advancing visual and ROI‐based quantitative tau staging (Figure 4). In K‐ROAD, visual and neo‐temporal and temporoparietal ROI‐based tau staging effectively differentiated cognitive decline between A+/Tmod+ and A+/Tadv+ as well as between A+/T− and A+/Tadv+. MTL‐based quantitative tau staging distinguished A+/T− from A+/Tmod+. In the ADNI cohort, both visual and quantitative tau staging effectively differentiated cognitive trajectories between A+/T− and A+/Tadv+, while all ROI‐based quantitative tau staging also distinguished A+/Tmod+ from A+/Tadv+. Additionally, for CDR‐SB, both visual and MTL‐based tau staging effectively differentiated A+/T− from A+/Tmod+ (Table S2 in supporting information).

## 4 DISCUSSION

In the present study, we compared visual assessments with various quantification methods for tau PET staging and evaluated plasma biomarkers and cognitive trajectories according to tau staging derived from these methods, leveraging two independent cohorts to enhance generalizability. Our major findings were as follows. First, among the quantification methods, the FreeSurfer without PVC achieved the highest AUC for differentiating visual tau positivity. Second, the meta‐temporal region demonstrated the best performance for early tau staging, while the temporoparietal region performed best for late tau staging. Third, plasma biomarker levels, especially p‐tau217, progressively increased across tau PET staging, with quantification methods outperforming visual assessment in distinguishing early or intermediate transitions, such as A+/T− to A+/Tmod+ or A+/Tmod+ to A+/Tadv+. Finally, quantification methods demonstrated advantages in differentiating cognitive decline across tau stages, particularly for intermediate transitions. However, visual assessments remained valuable, effectively distinguishing advanced tau pathology and, in some cases, early cognitive decline. Taken together, our findings demonstrate the complementary strengths of visual and quantification methods, enhancing tau PET staging by capturing biomarker changes and cognitive trajectories with greater precision. This integrated approach, validated across independent cohorts, not only improves diagnostic accuracy but also establishes a robust framework for monitoring disease progression and guiding personalized therapeutic strategies.

Our first major finding was that, among the quantification methods, the FreeSurfer without PVC and the FreeSurfer with PVC demonstrated the highest agreement with visual assessments, with the FreeSurfer without PVC achieving the highest AUC for differentiating tau positivity. Our findings align with previous studies showing that FreeSurfer often outperforms other methods, such as the MCALT method, in terms of diagnostic group separation, particularly when the cerebellum is used as the reference region.^48^ While previous studies focused on differentiating cognitively impaired and unimpaired individuals, our findings extend their utility to staging tau pathology. Notably, FreeSurfer without PVC showed enhanced performance compared to its PVC counterpart. Although PVC methods are widely used in AT PET studies to address variability due to atrophy and other factors,^51^, ^53^ they can introduce noise and artifacts, potentially reducing the precision of cortical SUVRs.^54^ These results suggest that, for tau pathology staging, avoiding PVC may yield more reliable outcomes when alignment with visual assessment is critical.

Our second major finding was that the meta‐temporal ROI demonstrated the best performance for early tau visual staging (T− vs. Tmod+), while the temporoparietal ROI was most effective for distinguishing late tau visual stages (Tmod+ vs. Tadv+). This aligns with the known progression of tau pathology from medial temporal areas to parietal and posterior cortical regions during advanced disease stages. The broader coverage of the meta‐temporal ROI, including inferior and middle temporal cortices, may explain its superior sensitivity in capturing early tau pathology, which visual assessments often miss due to their focus on more pronounced later‐stage changes.^7^ Conversely, the temporoparietal ROI's effectiveness in distinguishing moderate from advanced stages highlights the importance of region‐specific strategies tailored to disease stage when conducting tau PET staging.^55^, ^56^

Our third major finding was that plasma biomarker levels, especially p‐tau217, progressively increased across tau PET staging, with quantification methods outperforming visual assessment in detecting early and intermediate transitions such as A+/T− to A+/Tmod+ and A+/Tmod+ to A+/Tadv+. While both visual assessment and quantification methods effectively distinguished broader stages (e.g., A+/T− from A+/Tadv+), quantification methods demonstrated superior sensitivity in capturing subtle pathological changes. Notably, significant differences were observed in the MTL ROI between A+/T− and A+/Tmod+, while other ROIs (meta‐temporal, neo‐temporal, and temporoparietal) differentiated between A+/Tmod+ and A+/Tadv+. Visual assessment, primarily developed for diagnostic purposes, is reliable for detecting advanced tau pathology, as evidenced by a PET‐to‐autopsy study reporting 92% sensitivity and 80% specificity for Braak stages V and VI.^7^ However, this simplicity and practicality in clinical settings come with limitations, particularly in tracking early or intermediate transitions.^7^ Quantification methods, by providing greater granularity, complement visual assessment by enabling more precise monitoring of disease progression across all stages.

Our final major finding was that in K‐ROAD, quantitative tau staging (MTL, neo‐temporal, and temporoparietal ROIs) effectively differentiated cognitive decline across all stages (A+/T− vs. A+/Tmod+ vs. A+/Tadv+), whereas visual tau staging primarily distinguished cognitive decline between A+/T− and A+/Tadv+. Specifically, the MTL ROI effectively distinguished initial cognitive trajectories (A+/T− vs. A+/Tmod+), whereas the neo‐temporal and temporoparietal ROIs differentiated intermediate‐to‐advanced cognitive trajectories (A+/Tmod+ vs. A+/Tadv+), reflecting the expected relationship between ROI‐specific tau deposition and cognitive decline progression.^3^, ^24^, ^47^ In contrast, visual tau staging primarily distinguished cognitive decline between A+/T− and A+/Tadv+, suggesting it may be less sensitive to earlier disease transitions.^7^ Indeed, although visual criteria did not include the MTL for defining moderate positivity, quantitative tau staging based on the MTL ROI successfully captured differences in biomarker levels and cognitive trajectories between early stages (A+/T− vs. A+/Tmod+), highlighting the relevance of MTL tau deposition as an indicator of initial AD pathology. This limitation likely stems from the method's emphasis on cortical tau pathology, which effectively captures later‐stage accumulation but may not fully reflect early tau deposition in the MTL.^7^ Recognizing this, subsequent studies have proposed visual assessment approaches that incorporate the MTL, an early site of tau accumulation, to enhance sensitivity for detecting early tau pathology.^9^, ^11^, ^57^ Interestingly, findings in the ADNI cohort differed slightly—specifically, for CDR‐SB, visual tau staging effectively distinguished A+/T− from A+/Tmod+.^41^, ^58^ Whether this indicates that visual assessments can capture early cognitive trajectories under certain conditions or reflects cohort‐specific factors, such as demographic or genetic differences, warrants further investigation.^26^, ^59^, ^60^ Together, these results highlight the complementary strengths of visual assessment and quantification methods. While the FDA‐approved visual assessment provides a practical tool for detecting advanced tau pathology in clinical practice, it may miss subtler early‐stage tau accumulation, particularly in MTL regions. Quantitative methods can complement this limitation by providing enhanced sensitivity for detecting early and intermediate stages of tau pathology.

The strengths of the present study include its prospective setting, use of standardized AT PET and MRI protocols, and validation across two independent cohorts, enhancing the generalizability of findings. However, several limitations should be acknowledged. First, pathological verification was lacking. Although we proposed the presence of tau pathology defined by a “visual read” of a tau PET scan, we did not confirm its presence.^61^, ^62^ Second, quantitative approaches for tau PET may better capture early stages of tau deposition that are not identified using visual interpretation, highlighting the need for complementary use of both methods. Third, [^18^F]flortaucipir PET is known for off‐target binding in MTL areas (e.g., choroid plexus), potentially reducing specificity. Nonetheless, significant associations with early cognitive and biomarker changes suggest meaningful tau pathology is being captured despite this limitation. Fourth, the relatively limited number of participants in the cohort may have contributed to the absence of statistically significant differences in the rate of MMSE score changes between tau PET‐positive and ‐negative subjects across the target regions.^63^, ^64^ Fifth, while this study focused on cross‐sectional data, future research with longitudinal studies is essential to evaluate temporal changes in tau pathology progression and their relationship with clinical outcomes.^65^, ^66^ Despite these limitations, our study is notable for its systematic comparison of diverse methodologies to enhance tau pathology detection, emphasizing the importance of selecting appropriate methods and target regions. These findings underscore the complementary strengths of visual assessment and quantitative methods, offering practical insights for improving tau PET staging and guiding personalized therapeutic strategies.

In summary, our study highlights the complementary strengths of visual assessments and quantification methods for tau PET staging. By systematically comparing methods, ROIs, and their relationship with plasma biomarkers and cognitive trajectories, we demonstrated that quantification methods offer greater sensitivity in detecting intermediate transitions, while visual assessment provides simplicity and reliability for identifying advanced tau pathology. These findings advance our understanding of tau pathology progression and underscore the potential of integrating these approaches to enhance diagnostic precision and inform personalized therapeutic strategies.

## CONFLICT OF INTEREST STATEMENT

H.Z. has served on scientific advisory boards and/or as a consultant for Abbvie, Acumen, Alector, Alzinova, ALZPath, Amylyx, Annexon, Apellis, Artery Therapeutics, AZTherapies, Cognito Therapeutics, CogRx, Denali, Eisai, Merry Life, Nervgen, Novo Nordisk, Optoceutics, Passage Bio, Pinteon Therapeutics, Prothena, Red Abbey Labs, reMYND, Roche, Samumed, Siemens Healthineers, Triplet Therapeutics, and Wave; has delivered lectures in symposia sponsored by Alzecure, Biogen, Cellectricon, Fujirebio, Lilly, Novo Nordisk, and Roche; and is a co‐founder of Brain Biomarker Solutions in Gothenburg AB (BBS), which is a part of the GU Ventures Incubator Program (outside submitted work). K.B. has served as a consultant and was on advisory boards for Abbvie, AC Immune, ALZPath, AriBio, BioArctic, Biogen, Eisai, Lilly, Moleac Pte. Ltd, Novartis, Ono Pharma, Prothena, Roche Diagnostics, and Siemens Healthineers; has served on data monitoring committees for Julius Clinical and Novartis; has delivered lectures, produced educational materials, and participated in educational programs for AC Immune, Biogen, Celdara Medical, Eisai, and Roche Diagnostics; and is a co‐founder of Brain Biomarker Solutions in Gothenburg AB (BBS), which is a part of the GU Ventures Incubator Program, outside the work presented in this paper. M.S. has served/serves on advisory boards for Arvakor, Lilly, Novo Nordisk, and Roche; received speaker honoraria from Bioarctic, Novo Nordisk, Lilly, Roche, and Triolabs; and receives research support (to the institution) from Alzpath, Beckman Coulter, Bioarctic, Novo Nordisk, and Roche. He is co‐founder and shareholder of Centile Bioscience and serves as associate editor with *Alzheimer's Research & Therapy*. N.J.A. has received consulting fees from Quanterix, and has also received payments for lectures, presentations, speakers’ bureaus, manuscript writing, or educational events from Alamar Bio‐sciences, Biogen, Eli‐Lilly, and Quanterix. N.J.A. is listed as an inventor on a patent application (Application No.: PCT/US2024/037834, WSGR Docket No. 58484‐709.601) related to methods for remote blood collection, extraction, and analysis of neuro biomarkers; serves on the advisory board for Biogen, TargetALS, and TauRx; and receives payments for this role. B.H.B. reports that Korea Institute of Radiological & Medical Sciences (KIRAMS) entered into an exploratory investigator‐sponsored trial agreement with Avid Radiopharmaceuticals, Inc. on May 21, 2018, for the clinical study titled “Longitudinal Effects of Cerebrovascular and Amyloid Burdens on AV‐1451 Uptake” (agreement end date: December 31, 2027). Through this agreement, Avid Radiopharmaceuticals provided the precursor for 18F‐AV‐1451 and enabled its use but did not provide direct funding. Na and Seo are co‐founders of BeauBrain Healthcare, Inc. Other authors have no conflicts of interest to disclose.

## CONSENT STATEMENT

The institutional review board of Samsung Medical Center (No. 2021‐02_135) approved this study. All participants provided informed consent to participate in the study, and the data were collected in accordance with the Declaration of Helsinki.

## Supporting information



Supporting Information

Supporting Information
