# Supplementary material for: Integrating visual assessments and quantification methods for tau PET staging
Source: Alzheimers Dement. 2025 Jun 22;21(6):e70352. doi: 10.1002/alz.70352 (PMC12183112; doi:10.1002/alz.70352)
Supplement: Supplementary file 1 — Supporting Information [file ALZ-21-e70352-s001.docx]

**SUPPLEMENTARY MATERIAL**

**Table of contents**

**Supplementary Material 1. Plasma biomarker measurement**

**Supplementary Table S1. Alzheimer’s disease (AD) plasma biomarkers across AT stages**

**Supplementary Table S2. Cognitive trajectories across AT stages**

**Supplementary** **Figure 1. Study Flow Chart of Participant Inclusion and ExclusionSupplementary Matrial 1. Plasma biomarker measurement**

Blood samples (8 mL per participant) were collected in tubes containing 0.5 M EDTA and mixed for 5 minutes. Plasma was separated through centrifugation at 1300×g for 10 minutes and then aliquoted into five or ten vials, each containing 0.3 mL. All plasma samples were stored at -75 °C until analysis, following the National Biobank of the Republic of Korea’s guidelines for human resource collection and registration. The median time between plasma collection and Aβ PET scan was 4 days (interquartile range [IQR]: 0–69 days). For analysis, plasma samples were transported while frozen at -70 °C to the Department of Psychiatry and Neurochemistry at the University of Gothenburg. Before analysis, they were thawed on wet ice and centrifuged at 500×g for 5 minutes at 4 °C.

**Supplementary Table S1. Alzheimer’s disease (AD) plasma biomarkers across AT stages**

|  | **K-ROAD** | | | | | |  | **ADNI** | | | | | | |
| --- | --- | --- | --- | --- | --- | --- | --- | --- | --- | --- | --- | --- | --- | --- |
|  | **p-tau 217** | | **GFAP** | | **NFL** | |  | **p-tau 217** | | **GFAP** | | **NFL** | |  |
| **Method** | **Group Comparison** | ***p*-value^*^** | **Group Comparison** | ***p*-value** | **Group** | ***p*-value** | **Method** | **Group Comparison** | ***p*-value** | **Group Comparison** | ***p*-value** | **Group Comparison** | ***p*-value** |  |
| **VA** | A- vs. A+/T- | <0.001 | A- vs. A+/T- | 0.006 | A- vs. A+/T- | 0.827 | **VA** | A- vs. A+/T- | <0.001 | A- vs. A+/T- | 0.517 | A- vs. A+/T- | 0.910 |  |
|  | A- vs. A+/T_mod_+ | <0.001 | A- vs. A+/T_mod_+ | 0.221 | A- vs. A+/T_mod_+ | 0.434 |  | A- vs. A+/T_mod_+ | <0.001 | A- vs. A+/T_mod_+ | 0.070 | A- vs. A+/T_mod_+ | 0.145 |  |
|  | A- vs. A+/T_adv_+ | <0.001 | A- vs. A+/T_adv_+ | <0.001 | A- vs. A+/T_adv_+ | <0.001 |  | A- vs. A+/T_adv_+ | <0.001 | A- vs. A+/T_adv_+ | <0.001 | A- vs. A+/T_adv_+ | 0.004 |  |
|  | A+/T- vs. A+/T_mod_+ | 0.577 | A+/T- vs. A+/T_mod_+ | 0.956 | A+/T- vs. A+/T_mod_+ | 0.788 |  | A+/T- vs. A+/T_mod_+ | 0.140 | A+/T- vs. A+/T_mod_+ | 0.232 | A+/T- vs. A+/T_mod_+ | 0.255 |  |
|  | A+/T- vs. A+/T_adv_+ | <0.001 | A+/T- vs. A+/T_adv_+ | 0.046 | A+/T- vs. A+/T_adv_+ | 0.010 |  | A+/T- vs. A+/T_adv_+ | <0.001 | A+/T- vs. A+/T_adv_+ | <0.001 | A+/T- vs. A+/T_adv_+ | 0.040 |  |
|  | A+/T_mod_+ vs. A+/T_adv_+ | 0.495 | A+/T_mod_+ vs. A+/T_adv_+ | 0.121 | A+/T_mod_+ vs. A+/T_adv_+ | 0.737 |  | A+/T_mod_+ vs. A+/T_adv_+ | 0.630 | A+/T_mod_+ vs. A+/T_adv_+ | 0.999 | A+/T_mod_+ vs. A+/T_adv_+ | 0.953 |  |
| **MTL** | A- vs. A+/T- | <0.001 | A- vs. A+/T- | 0.006 | A- vs. A+/T- | 0.616 | **MTL** | A- vs. A+/T- | <0.001 | A- vs. A+/T- | 0.995 | A- vs. A+/T- | 1.000 |  |
|  | A- vs. A+/T_mod_+ | <0.001 | A- vs. A+/T_mod_+ | <0.001 | A- vs. A+/T_mod_+ | 0.005 |  | A- vs. A+/T_mod_+ | <0.001 | A- vs. A+/T_mod_+ | <0.001 | A- vs. A+/T_mod_+ | 0.024 |  |
|  | A- vs. A+/T_adv_+ | <0.001 | A- vs. A+/T_adv_+ | <0.001 | A- vs. A+/T_adv_+ | < 0.001 |  | A- vs. A+/T_adv_+ | <0.001 | A- vs. A+/T_adv_+ | <0.001 | A- vs. A+/T_adv_+ | 0.017 |  |
|  | A+/T- vs. A+/T_mod_+ | 0.002 | A+/T- vs. A+/T_mod_+ | 0.199 | A+/T- vs. A+/T_mod_+ | 0.134 |  | A+/T- vs. A+/T_mod_+ | <0.001 | A+/T- vs. A+/T_mod_+ | 0.001 | A+/T- vs. A+/T_mod_+ | 0.073 |  |
|  | A+/T- vs. A+/T_adv_+ | <0.001 | A+/T- vs. A+/T_adv_+ | 0.022 | A+/T- vs. A+/T_adv_+ | 0.041 |  | A+/T- vs. A+/T_adv_+ | <0.001 | A+/T- vs. A+/T_adv_+ | 0.001 | A+/T- vs. A+/T_adv_+ | 0.050 |  |
|  | A+/T_mod_+ vs. A+/T_adv_+ | 0.193 | A+/T_mod_+ vs. A+/T_adv_+ | 0.426 | A+/T_mod_+ vs. A+/T_adv_+ | 0.725 |  | A+/T_mod_+ vs. A+/T_adv_+ | 0.300 | A+/T_mod_+ vs. A+/T_adv_+ | 0.995 | A+/T_mod_+ vs. A+/T_adv_+ | 0.999 |  |
| **Meta** | A- vs. A+/T- | <0.001 | A- vs. A+/T- | 0.011 | A- vs. A+/T- | 0.874 | **Meta** | A- vs. A+/T- | <0.001 | A- vs. A+/T- | 0.527 | A- vs. A+/T- | 0.966 |  |
|  | A- vs. A+/T_mod_+ | <0.001 | A- vs. A+/T_mod_+ | 0.006 | A- vs. A+/T_mod_+ | 0.013 |  | A- vs. A+/T_mod_+ | <0.001 | A- vs. A+/T_mod_+ | 0.267 | A- vs. A+/T_mod_+ | 0.661 |  |
|  | A- vs. A+/T_adv_+ | <0.001 | A- vs. A+/T_adv_+ | <0.001 | A- vs. A+/T_adv_+ | < 0.001 |  | A- vs. A+/T_adv_+ | <0.001 | A- vs. A+/T_adv_+ | < 0.001 | A- vs. A+/T_adv_+ | < 0.001 |  |
|  | A+/T- vs. A+/T_mod_+ | 0.279 | A+/T- vs. A+/T_mod_+ | 0.931 | A+/T- vs. A+/T_mod_+ | 0.057 |  | A+/T- vs. A+/T_mod_+ | 0.679 | A+/T- vs. A+/T_mod_+ | 0.915 | A+/T- vs. A+/T_mod_+ | 0.903 |  |
|  | A+/T- vs. A+/T_adv_+ | <0.001 | A+/T- vs. A+/T_adv_+ | <0.001 | A+/T- vs. A+/T_adv_+ | 0.003 |  | A+/T- vs. A+/T_adv_+ | <0.001 | A+/T- vs. A+/T_adv_+ | < 0.001 | A+/T- vs. A+/T_adv_+ | 0.004 |  |
|  | A+/T_mod_+ vs. A+/T_adv_+ | 0.023 | A+/T_mod_+ vs. A+/T_adv_+ | 0.040 | A+/T_mod_+ vs. A+/T_adv_+ | 0.999 |  | A+/T_mod_+ vs. A+/T_adv_+ | <0.001 | A+/T_mod_+ vs. A+/T_adv_+ | 0.004 | A+/T_mod_+ vs. A+/T_adv_+ | 0.073 |  |
| **NEO** | A- vs. A+/T- | <0.001 | A- vs. A+/T- | 0.125 | A- vs. A+/T- | 0.931 | **NEO** | A- vs. A+/T- | <0.001 | A- vs. A+/T- | 0.824 | A- vs. A+/T- | 0.959 |  |
|  | A- vs. A+/T_mod_+ | <0.001 | A- vs. A+/T_mod_+ | <0.001 | A- vs. A+/T_mod_+ | 0.040 |  | A- vs. A+/T_mod_+ | <0.001 | A- vs. A+/T_mod_+ | 0.099 | A- vs. A+/T_mod_+ | 0.767 |  |
|  | A- vs. A+/T_adv_+ | <0.001 | A- vs. A+/T_adv_+ | <0.001 | A- vs. A+/T_adv_+ | < 0.001 |  | A- vs. A+/T_adv_+ | <0.001 | A- vs. A+/T_adv_+ | < 0.001 | A- vs. A+/T_adv_+ | < 0.001 |  |
|  | A+/T- vs. A+/T_mod_+ | 0.077 | A+/T- vs. A+/T_mod_+ | 0.027 | A+/T- vs. A+/T_mod_+ | 0.159 |  | A+/T- vs. A+/T_mod_+ | 0.751 | A+/T- vs. A+/T_mod_+ | 0.546 | A+/T- vs. A+/T_mod_+ | 0.969 |  |
|  | A+/T- vs. A+/T_adv_+ | <0.001 | A+/T- vs. A+/T_adv_+ | <0.001 | A+/T- vs. A+/T_adv_+ | 0.002 |  | A+/T- vs. A+/T_adv_+ | <0.001 | A+/T- vs. A+/T_adv_+ | < 0.001 | A+/T- vs. A+/T_adv_+ | 0.003 |  |
|  | A+/T_mod_+ vs. A+/T_adv_+ | 0.020 | A+/T_mod_+ vs. A+/T_adv_+ | 0.263 | A+/T_mod_+ vs. A+/T_adv_+ | 0.362 |  | A+/T_mod_+ vs. A+/T_adv_+ | <0.001 | A+/T_mod_+ vs. A+/T_adv_+ | 0.005 | A+/T_mod_+ vs. A+/T_adv_+ | 0.027 |  |
| **TP** | A- vs. A+/T- | <0.001 | A- vs. A+/T- | 0.074 | A- vs. A+/T- | 0.855 | **TP** | A- vs. A+/T- | < 0.001 | A- vs. A+/T- | 0.436 | A- vs. A+/T- | 0.790 |  |
|  | A- vs. A+/T_mod_+ | <0.001 | A- vs. A+/T_mod_+ | <0.001 | A- vs. A+/T_mod_+ | 0.032 |  | A- vs. A+/T_mod_+ | < 0.001 | A- vs. A+/T_mod_+ | 0.180 | A- vs. A+/T_mod_+ | 0.795 |  |
|  | A- vs. A+/T_adv_+ | <0.001 | A- vs. A+/T_adv_+ | <0.001 | A- vs. A+/T_adv_+ | < 0.001 |  | A- vs. A+/T_adv_+ | < 0.001 | A- vs. A+/T_adv_+ | < 0.001 | A- vs. A+/T_adv_+ | 0.002 |  |
|  | A+/T- vs. A+/T_mod_+ | 0.126 | A+/T- vs. A+/T_mod_+ | 0.059 | A+/T- vs. A+/T_mod_+ | 0.179 |  | A+/T- vs. A+/T_mod_+ | 1.000 | A+/T- vs. A+/T_mod_+ | 0.864 | A+/T- vs. A+/T_mod_+ | 0.998 |  |
|  | A+/T- vs. A+/T_adv_+ | <0.001 | A+/T- vs. A+/T_adv_+ | <0.001 | A+/T- vs. A+/T_adv_+ | 0.005 |  | A+/T- vs. A+/T_adv_+ | < 0.001 | A+/T- vs. A+/T_adv_+ | 0.002 | A+/T- vs. A+/T_adv_+ | 0.070 |  |
|  | A+/T_mod_+ vs. A+/T_adv_+ | 0.016 | A+/T_mod_+ vs. A+/T_adv_+ | 0.256 | A+/T_mod_+ vs. A+/T_adv_+ | 0.591 |  | A+/T_mod_+ vs. A+/T_adv_+ | 0.005 | A+/T_mod_+ vs. A+/T_adv_+ | 0.090 | A+/T_mod_+ vs. A+/T_adv_+ | 0.218 |  |

**p*-value from Tukey-corrected post hoc tests

The plasma biomarker levels between the groups were assessed using analysis of covariance (ANCOVA), with age and *APOE* genotypes as covariates. Abbreviations: K-ROAD, Korea-Registries to Overcome dementia and Accelerate Dementia; ADNI, Alzheimer's Disease Neuroimaging Initiative; A, amyloid; T, tau; mod, moderate; adv, advanced; VA, visual assessments; MTL, medial temporal lobe; Meta, temporal meta-region; NEO, neo-temporal; TP, temporoparietal regions; p-tau217, phosphorylated-tau 217; GFAP, glial fibrillary acidic protein; NfL, neurofilament light chain.

**Supplementary Table S2. Cognitive trajectories across AT stages**

|  | **K-ROAD** | | | | | |  | **ADNI** | | | | | | |
| --- | --- | --- | --- | --- | --- | --- | --- | --- | --- | --- | --- | --- | --- | --- |
| **Method** | **Outcome** | **Group Comparison** | **beta** | **se** | ***p*-value** | **adjusted *p*-value^*^** | **Method** | **Outcome** | **Group Comparison** | **beta** | **se** | ***p*-value** | **adjusted *p*-value** |  |
| **VA** | **MMSE** |  |  |  |  |  | **VA** | **MMSE** |  |  |  |  |  |  |
|  |  | A- vs. A+/T- | -0.397 | 0.266 | 0.138 | 0.138 |  |  | A- vs. A+/T- | 0.009 | 0.057 | 0.869 | 0.579 |  |
|  |  | A- vs. A+/T_mod_+ | -0.140 | 0.396 | 0.723 | 0.482 |  |  | A- vs. A+/T_mod_+ | -0.171 | 0.116 | 0.142 | 0.114 |  |
|  |  | A- vs. A+/T_adv_+ | -1.757 | 0.215 | <0.001 | <0.001 |  |  | A- vs. A+/T_adv_+ | -0.580 | 0.060 | <0.001 | <0.001 |  |
|  |  | A+/T- vs. A+/T_mod_+ | 0.257 | 0.399 | 0.521 | 0.417 |  |  | A+/T- vs. A+/T_mod_+ | -0.181 | 0.121 | 0.138 | 0.138 |  |
|  |  | A+/T- vs. A+/T_adv_+ | -1.360 | 0.222 | 0.000 | 0.000 |  |  | A+/T- vs. A+/T_adv_+ | -0.589 | 0.070 | <0.001 | <0.001 |  |
|  |  | A+/T_mod_+ vs. A+/T_adv_+ | -1.616 | 0.368 | <0.001 | <0.001 |  |  | A+/T_mod_+ vs. A+/T_adv_+ | -0.408 | 0.123 | 0.001 | 0.001 |  |
|  | **CDR_SB** |  |  |  |  |  |  | **CDR_SB** |  |  |  |  |  |  |
|  |  | A- vs. A+/T- | 0.153 | 0.144 | 0.291 | 0.291 |  |  | A- vs. A+/T- | 0.028 | 0.041 | 0.491 | 0.327 |  |
|  |  | A- vs. A+/T_mod_+ | 0.154 | 0.225 | 0.494 | 0.396 |  |  | A- vs. A+/T_mod_+ | 0.197 | 0.084 | 0.019 | 0.019 |  |
|  |  | A- vs. A+/T_adv_+ | 0.861 | 0.119 | <0.001 | <0.001 |  |  | A- vs. A+/T_adv_+ | 0.469 | 0.043 | 0.000 | 0.000 |  |
|  |  | A+/T- vs. A+/T_mod_+ | 0.001 | 0.229 | 0.996 | 0.664 |  |  | A+/T- vs. A+/T_mod_+ | 0.169 | 0.088 | 0.054 | 0.043 |  |
|  |  | A+/T- vs. A+/T_adv_+ | 0.709 | 0.128 | <0.001 | <0.001 |  |  | A+/T- vs. A+/T_adv_+ | 0.441 | 0.050 | <0.001 | <0.001 |  |
|  |  | A+/T_mod_+ vs. A+/T_adv_+ | 0.708 | 0.215 | 0.001 | 0.002 |  |  | A+/T_mod_+ vs. A+/T_adv_+ | 0.272 | 0.089 | 0.002 | 0.003 |  |
| **MTL** | **MMSE** |  |  |  |  |  | **MTL** | **MMSE** |  |  |  |  |  |  |
|  |  | A- vs. A+/T- | -0.470 | 0.286 | 0.102 | 0.068 |  |  | A- vs. A+/T- | 0.032 | 0.063 | 0.605 | 0.403 |  |
|  |  | A- vs. A+/T_mod_+ | -1.402 | 0.241 | <0.001 | <0.001 |  |  | A- vs. A+/T_mod_+ | -0.140 | 0.064 | 0.029 | 0.023 |  |
|  |  | A- vs. A+/T_adv_+ | -1.895 | 0.268 | <0.001 | <0.001 |  |  | A- vs. A+/T_adv_+ | -0.707 | 0.066 | 0.000 | 0.000 |  |
|  |  | A+/T- vs. A+/T_mod_+ | -0.932 | 0.251 | <0.001 | <0.001 |  |  | A+/T- vs. A+/T_mod_+ | -0.172 | 0.078 | 0.028 | 0.028 |  |
|  |  | A+/T- vs. A+/T_adv_+ | -1.425 | 0.277 | <0.001 | <0.001 |  |  | A+/T- vs. A+/T_adv_+ | -0.739 | 0.080 | <0.001 | <0.001 |  |
|  |  | A+/T_mod_+ vs. A+/T_adv_+ | -0.492 | 0.231 | 0.035 | 0.028 |  |  | A+/T_mod_+ vs. A+/T_adv_+ | -0.567 | 0.081 | <0.001 | <0.001 |  |
|  | **CDR_SB** |  |  |  |  |  |  | **CDR_SB** |  |  |  |  |  |  |
|  |  | A- vs. A+/T- | 0.156 | 0.152 | 0.307 | 0.205 |  |  | A- vs. A+/T- | 0.011 | 0.045 | 0.817 | 0.545 |  |
|  |  | A- vs. A+/T_mod_+ | 0.631 | 0.127 | <0.001 | <0.001 |  |  | A- vs. A+/T_mod_+ | 0.190 | 0.047 | <0.001 | <0.001 |  |
|  |  | A- vs. A+/T_adv_+ | 1.024 | 0.152 | <0.001 | <0.001 |  |  | A- vs. A+/T_adv_+ | 0.527 | 0.048 | <0.001 | <0.001 |  |
|  |  | A+/T- vs. A+/T_mod_+ | 0.474 | 0.141 | 0.001 | 0.001 |  |  | A+/T- vs. A+/T_mod_+ | 0.179 | 0.057 | 0.002 | 0.001 |  |
|  |  | A+/T- vs. A+/T_adv_+ | 0.867 | 0.163 | <0.001 | <0.001 |  |  | A+/T- vs. A+/T_adv_+ | 0.516 | 0.058 | <0.001 | <0.001 |  |
|  |  | A+/T_mod_+ vs. A+/T_adv_+ | 0.393 | 0.141 | 0.006 | 0.005 |  |  | A+/T_mod_+ vs. A+/T_adv_+ | 0.337 | 0.059 | <0.001 | <0.001 |  |
| **Meta** | **MMSE** |  |  |  |  |  | **Meta** | **MMSE** |  |  |  |  |  |  |
|  |  | A- vs. A+/T- | -0.402 | 0.256 | 0.119 | 0.095 |  |  | A- vs. A+/T- | -0.001 | 0.060 | 0.982 | 0.655 |  |
|  |  | A- vs. A+/T_mod_+ | -0.492 | 0.294 | 0.097 | 0.097 |  |  | A- vs. A+/T_mod_+ | -0.068 | 0.066 | 0.306 | 0.306 |  |
|  |  | A- vs. A+/T_adv_+ | -1.898 | 0.212 | <0.001 | <0.001 |  |  | A- vs. A+/T_adv_+ | -0.735 | 0.065 | <0.001 | <0.001 |  |
|  |  | A+/T- vs. A+/T_mod_+ | -0.090 | 0.297 | 0.763 | 0.509 |  |  | A+/T- vs. A+/T_mod_+ | -0.066 | 0.078 | 0.397 | 0.318 |  |
|  |  | A+/T- vs. A+/T_adv_+ | -1.496 | 0.217 | <0.001 | <0.001 |  |  | A+/T- vs. A+/T_adv_+ | -0.733 | 0.077 | <0.001 | <0.001 |  |
|  |  | A+/T_mod_+ vs. A+/T_adv_+ | -1.407 | 0.261 | <0.001 | <0.001 |  |  | A+/T_mod_+ vs. A+/T_adv_+ | -0.667 | 0.082 | <0.001 | <0.001 |  |
|  | **CDR_SB** |  |  |  |  |  |  | **CDR_SB** |  |  |  |  |  |  |
|  |  | A- vs. A+/T- | 0.125 | 0.134 | 0.352 | 0.282 |  |  | A- vs. A+/T- | 0.033 | 0.044 | 0.446 | 0.297 |  |
|  |  | A- vs. A+/T_mod_+ | 0.180 | 0.163 | 0.272 | 0.272 |  |  | A- vs. A+/T_mod_+ | 0.147 | 0.048 | 0.002 | 0.002 |  |
|  |  | A- vs. A+/T_adv_+ | 0.959 | 0.113 | 0.000 | 0.000 |  |  | A- vs. A+/T_adv_+ | 0.548 | 0.047 | <0.001 | <0.001 |  |
|  |  | A+/T- vs. A+/T_mod_+ | 0.054 | 0.169 | 0.748 | 0.499 |  |  | A+/T- vs. A+/T_mod_+ | 0.113 | 0.057 | 0.048 | 0.038 |  |
|  |  | A+/T- vs. A+/T_adv_+ | 0.834 | 0.122 | <0.001 | <0.001 |  |  | A+/T- vs. A+/T_adv_+ | 0.514 | 0.056 | <0.001 | <0.001 |  |
|  |  | A+/T_mod_+ vs. A+/T_adv_+ | 0.780 | 0.154 | <0.001 | <0.001 |  |  | A+/T_mod_+ vs. A+/T_adv_+ | 0.401 | 0.059 | <0.001 | <0.001 |  |
| **NEO** | **MMSE** |  |  |  |  |  | **NEO** | **MMSE** |  |  |  |  |  |  |
|  |  | A- vs. A+/T- | -0.220 | 0.244 | 0.369 | 0.246 |  |  | A- vs. A+/T- | -0.029 | 0.063 | 0.644 | 0.515 |  |
|  |  | A- vs. A+/T_mod_+ | -0.704 | 0.219 | 0.002 | 0.002 |  |  | A- vs. A+/T_mod_+ | -0.035 | 0.061 | 0.568 | 0.568 |  |
|  |  | A- vs. A+/T_adv_+ | -2.114 | 0.197 | 0.000 | 0.000 |  |  | A- vs. A+/T_adv_+ | -0.766 | 0.066 | <0.001 | <0.001 |  |
|  |  | A+/T- vs. A+/T_mod_+ | -0.484 | 0.238 | 0.044 | 0.035 |  |  | A+/T- vs. A+/T_mod_+ | -0.006 | 0.076 | 0.939 | 0.626 |  |
|  |  | A+/T- vs. A+/T_adv_+ | -1.894 | 0.217 | <0.001 | <0.001 |  |  | A+/T- vs. A+/T_adv_+ | -0.737 | 0.080 | <0.001 | <0.001 |  |
|  |  | A+/T_mod_+ vs. A+/T_adv_+ | -1.410 | 0.189 | <0.001 | <0.001 |  |  | A+/T_mod_+ vs. A+/T_adv_+ | -0.731 | 0.079 | <0.001 | <0.001 |  |
|  | **CDR_SB** |  |  |  |  |  |  | **CDR_SB** |  |  |  |  |  |  |
|  |  | A- vs. A+/T- | 0.097 | 0.132 | 0.465 | 0.310 |  |  | A- vs. A+/T- | 0.059 | 0.046 | 0.199 | 0.159 |  |
|  |  | A- vs. A+/T_mod_+ | 0.302 | 0.125 | 0.018 | 0.018 |  |  | A- vs. A+/T_mod_+ | 0.115 | 0.045 | 0.011 | 0.011 |  |
|  |  | A- vs. A+/T_adv_+ | 1.088 | 0.113 | 0.000 | 0.000 |  |  | A- vs. A+/T_adv_+ | 0.562 | 0.048 | <0.001 | <0.001 |  |
|  |  | A+/T- vs. A+/T_mod_+ | 0.205 | 0.139 | 0.143 | 0.114 |  |  | A+/T- vs. A+/T_mod_+ | 0.056 | 0.056 | 0.323 | 0.215 |  |
|  |  | A+/T- vs. A+/T_adv_+ | 0.991 | 0.128 | <0.001 | <0.001 |  |  | A+/T- vs. A+/T_adv_+ | 0.503 | 0.058 | <0.001 | <0.001 |  |
|  |  | A+/T_mod_+ vs. A+/T_adv_+ | 0.786 | 0.120 | <0.001 | <0.001 |  |  | A+/T_mod_+ vs. A+/T_adv_+ | 0.447 | 0.058 | <0.001 | <0.001 |  |
| **TP** | **MMSE** |  |  |  |  |  | **TP** | **MMSE** |  |  |  |  |  |  |
|  |  | A- vs. A+/T- | -0.242 | 0.229 | 0.293 | 0.196 |  |  | A- vs. A+/T- | -0.039 | 0.060 | 0.521 | 0.521 |  |
|  |  | A- vs. A+/T_mod_+ | -0.737 | 0.220 | 0.001 | 0.001 |  |  | A- vs. A+/T_mod_+ | -0.020 | 0.069 | 0.771 | 0.616 |  |
|  |  | A- vs. A+/T_adv_+ | -2.154 | 0.195 | 0.000 | 0.000 |  |  | A- vs. A+/T_adv_+ | -0.700 | 0.065 | <0.001 | <0.001 |  |
|  |  | A+/T- vs. A+/T_mod_+ | -0.495 | 0.227 | 0.032 | 0.025 |  |  | A+/T- vs. A+/T_mod_+ | 0.018 | 0.081 | 0.819 | 0.546 |  |
|  |  | A+/T- vs. A+/T_adv_+ | -1.912 | 0.203 | <0.001 | <0.001 |  |  | A+/T- vs. A+/T_adv_+ | -0.662 | 0.077 | <0.001 | <0.001 |  |
|  |  | A+/T_mod_+ vs. A+/T_adv_+ | -1.417 | 0.192 | <0.001 | <0.001 |  |  | A+/T_mod_+ vs. A+/T_adv_+ | -0.680 | 0.084 | <0.001 | <0.001 |  |
|  | **CDR_SB** |  |  |  |  |  |  | **CDR_SB** |  |  |  |  |  |  |
|  |  | A- vs. A+/T- | 0.088 | 0.119 | 0.459 | 0.306 |  |  | A- vs. A+/T- | 0.094 | 0.044 | 0.033 | 0.033 |  |
|  |  | A- vs. A+/T_mod_+ | 0.364 | 0.119 | 0.003 | 0.003 |  |  | A- vs. A+/T_mod_+ | 0.069 | 0.051 | 0.174 | 0.139 |  |
|  |  | A- vs. A+/T_adv_+ | 1.143 | 0.109 | 0.000 | 0.000 |  |  | A- vs. A+/T_adv_+ | 0.524 | 0.047 | 0.000 | 0.000 |  |
|  |  | A+/T- vs. A+/T_mod_+ | 0.276 | 0.127 | 0.032 | 0.025 |  |  | A+/T- vs. A+/T_mod_+ | -0.025 | 0.059 | 0.667 | 0.445 |  |
|  |  | A+/T- vs. A+/T_adv_+ | 1.054 | 0.118 | <0.001 | <0.001 |  |  | A+/T- vs. A+/T_adv_+ | 0.430 | 0.056 | <0.001 | <0.001 |  |
|  |  | A+/T_mod_+ vs. A+/T_adv_+ | 0.779 | 0.118 | <0.001 | <0.001 |  |  | A+/T_mod_+ vs. A+/T_adv_+ | 0.455 | 0.061 | <0.001 | <0.001 |  |

**p*-value adjusted for FDR correction

The cognitive trajectories across the four groups using linear mixed-effect models with fixed effects including age, *APOE* ε4 carrier status, group, time, and group by time interaction term. Abbreviations: K-ROAD, Korea-Registries to Overcome dementia and Accelerate Dementia; ADNI, Alzheimer's Disease Neuroimaging Initiative; A, amyloid; T, tau; mod, moderate; adv, advanced; VA, visual assessments; MTL; medial temporal lobe; Meta, temporal meta-region; NEO, neo-temporal; TP, temporoparietal regions; MMSE, Mini-Mental State Examination; CDR-SB, Clinical Dementia Rating Scale-Sum of Boxes.

**Supplementary Figure**

**K-ROAD ADNI**

**Cross-sectional analyses**

Dx: CU/MCI/AD-D
(N=870)

Dx: CU/MCI/AD-D/SVCI/FTD (N=289)

Excluded: SVCI/FTD (N=0)

Excluded: SVCI/FTD (N=115)

Excluded: Missing longitudinal MMSE
(N=10)

**Longitudinal analyses**

Dx : CU/MCI/AD-D
(N=164)

Dx : CU/MCI/AD-D
(N=870)

**Supplementary Figure 1: Study Flow Chart of Participant Inclusion and Exclusion.** Participants diagnosed with SVCI or FTD were included in the initial cross-sectional analyses but excluded from longitudinal analyses to ensure homogeneous samples consisting only CU, MCI, and AD-D participants. Abbreviations: K-ROAD, Korea-Registries to Overcome dementia and Accelerate Dementia; ADNI, Alzheimer's Disease Neuroimaging Initiative; Dx, Diagnoses; CU, Cognitively Unimpaired; MCI, Mild Cognitive Impairment; AD-D, Alzheimer’s disease dementia type; SVCI, Subcortical Vascular Cognitive Impairment; FTD, Frontotemporal Dementia; MMSE, Mini-Mental State Examination.
